# Characterization of cells resistant to the potent histone deacetylase inhibitor spiruchostatin B (SP-B) and effect of overexpressed p21^waf1/cip1^ on the SP-B resistance or susceptibility of human leukemia cells

**DOI:** 10.3892/ijo.2012.1507

**Published:** 2012-06-06

**Authors:** SYU-ICHI KANNO, NAOYUKI MAEDA, AYAKO TOMIZAWA, SHIN YOMOGIDA, TADASHI KATOH, MASAAKI ISHIKAWA

**Affiliations:** 1 Department of Clinical Pharmacotherapeutics;; 2 Laboratory of Synthetic Medicinal Chemistry, Department of Chemical Pharmaceutical Science, Tohoku Pharmaceutical University, Aoba-ku, Sendai 981-8558, Japan

**Keywords:** spiruchostatin, histone deacetylase inhibitor, p21^waf1/cip1^, drug resistant, apoptosis

## Abstract

We previously showed that the B cell leukemia cell line NALM-6 had the highest susceptibility among a number of leukemia cell lines to spiruchostatin B (SP-B), a potent histone deacetylase (HDAC) inhibitor. We also showed that SP-B-induced cytotoxicity depended on induction of apoptosis that was mediated by p21^waf1/cip1^ expression. In the present study, we generated and characterized a stable, SP-B-resistant NALM-6 cell line (NALM-6/SP-B) by continuous exposure to SP-B, starting with a low SP-B concentration. NALM-6/SP-B cells were also more resistant to FK228, which has a similar chemical structure to SP-B, and were slightly more resistant to the P-gp substrates doxorubicin and vincristine than parental cells, but displayed similar susceptibility to other HDAC inhibitors and to paclitaxel as the parental cells. There was little change in the basal mRNA expression of *HDAC1*, *p53*, *Bax*, *Bcl-2*, *Fas*, *caspase-3*, *c-Myc* and *MDR1* in NALM-6/SP-B compared to parental cells, but the mRNA expression of *p21^waf1/cip1^* was decreased. The introduction of an exogenous p21^waf1/cip1^ expression vector restored SP-B induction of NALM-6/SP-B cell apoptosis. Moreover, overexpressed p21^waf1/cip1^ enhanced SP-B induction of the apoptosis of the human erythroleukemia leukemia cell line K562 which is less susceptible to SP-B than NALM-6 cells. These results suggest that downregulation of *p21^waf1/cip1^*, which is a characteristic feature of NALM-6/SP-B cells, was important for their resistance to SP-B, and that this SP-B resistance could be overcome by the introduction of exogenous p21^waf1/cip1^. Furthermore, introduction of p21^waf1/cip1^ to other leukemia cells such as K562 may enhance their susceptibility to SP-B. This is the first report of the characterization of SP-B-resistant cells and of the effect of overexpressed p21^waf1/cip1^ on the resistance or susceptibility of human leukemia cells to SP-B.

## Introduction

The acetylation status of histones plays an important role in the regulation of gene expression by altering chromatin structure. Histone deacetylases (HDAC) facilitate a closed chromatin structure and hence transcriptional repression. HDAC function is commonly affected in human cancers. Therefore, inhibition of HDAC represents a novel therapeutic approach for cancer therapy. In our previous study, we compared the activity of structurally similar bicyclic depsipeptide compounds using a panel of 39 human cancer cell lines and found that spiruchostatin B (SP-B) was the most potent inhibitor of HDAC1 and displayed the most potent growth-inhibitory activity (1).

The anti-tumor effects of HDAC inhibitors (HDIs) have been shown to be mediated by various signaling mechanisms. One such mechanism involves release of HDAC1 from its binding to the p21^waf1/cip1^ promoter, including to the Sp1 sites on this promoter, which results in enhanced histone acetylation around these promoter sites and strong activation of the expression of the cyclin-dependent kinase inhibitor p21^waf1/cip1^(2). p21^waf1/cip1^ was initially identified as a cell cycle regulatory protein that can cause cell cycle arrest (3). Therefore, p21^waf1/cip1^ is a key gene expression target of HDI anti-tumor activity, and induction of p21^waf1/cip1^ by inhibition of HDAC function is critical for HDI blockage of cell cycle progression (4–7). Indeed, we have shown that NALM-6, a B cell leukemia cell line that has higher expression of *p21^waf1/cip1^* mRNA compared with other typical leukemia cell lines, was susceptible to SP-B-induced cytotoxicity that depended on induction of apoptosis (8). This result suggests that the introduction of exogenous p21^waf1/cip1^ into human leukemia cells that have a low susceptibility to SP-B will enhance their susceptibility to SP-B.

There are several major problems with the use of anti-cancer drugs for the chemotherapy of cancer patients. One such problem is the acquisition of drug resistance by tumor cells. Development of resistance to chemotherapy is therefore a major concern with any new therapy. It is well established that one mechanism of resistance to chemotherapeutic agents involves the expression of MDR1 (P-glycoprotein, P-gp), which can induce increased efflux of anticancer agents from tumor cells (9). P-gp is a major contributor to the resistance of cancer cells to FK228, a chemical with a similar structure to that of SP-B (10–12). It has also been shown that treatment with other HDIs such as suberoylanilide hydroxamic acid (SAHA, vorinostat) can result in the acquisition of irreversible and multidrug resistance-independent HDI resistance in HCT colon tumor cells (13,14). To date, there has been no report on the establishment of an SP-B-resistant human leukemia cell line, characterization of such an SP-B-resistant cell line or determination of what molecule might reverse such resistance.

In the present study, we generated and characterized a stable, SP-B-resistant NALM-6 cell (NALM-6/SP-B) line by continuous exposure of the cells to SP-B, starting with a low concentration. We also tested whether introduction of exogenous p21^waf1/cip1^ would reverse the SP-B resistance of NALM-6/SP-B cells, or enhance the susceptibility of the K562 human erythroleukemia leukemia cell line, which is less susceptible to SP-B than NALM-6 cells, to SP-B-induced apoptosis. The ultimate aims of this study were to better understand the acquisition of resistance or susceptibility to SP-B with a view of its clinical application for the chemotherapy of leukemia.

## Materials and methods

### Materials and cell culture

SP-B was prepared as previously described (1,15). Trichostatin A (TSA), sodium butyrate (NaB) and all other reagents, unless stated, were of the highest grade available and were supplied by either Sigma (St. Louis, MO, USA) or Wako Pure Chemical Industries, Ltd (Osaka, Japan). All cells were supplied by the Cell Resource Center for Biomedical Research, Tohoku University (Sendai, Japan). Cells were routinely cultured using standard methods as described in our previous report (16).

### Cytotoxicity and apoptosis

Cytotoxicity was assessed using the MTT (3-(4,5-dimethylthiazol-2-yl)-2,5-diphenyl tetrazolium bromide) assay, and apoptosis was estimated based on nuclear morphological observation using by our previously described method (17).

### Establishment of a spiruchostatin B-resistant cell line, NALM-6/SP-B

NALM-6/SP-B cells were obtained using a modification of a method for obtaining Ara-C-resistant cells (17). NALM-6 parental cells were cultured in increasing concentrations of SP-B, starting at 0.1 nM. Viable cells were then passaged into a higher concentration of NALM-6 in 0.1 nM increments until a concentration of 6 nM SP-B was reached. NALM-6/SP-B cells were then maintained in complete RPMI-1640 medium containing 6 nM SP-B.

### RNA isolation and quantitative real-time polymerase chain reaction (qPCR) assay

The mRNA expression level of *p21^waf1/cip1^* (GenBank Accession no. NM_000389.4), *HDAC1* (NM_004964.2), *p53* (NM_000546.4), *Bax* (NM_138761.3), *Bcl-2* (NM_000633.2), *Fas* (NM_000043.3), *caspase-3* (*Cas-3*, NM_004346.3), *c-Myc* (NM_002467.3), or *multidrug resistance* (*MDR1*, NM_000927.3) in NALM-6 or NALM-6/SP-B cells was quantified using the real-time polymerase chain reaction (qPCR) with a Light Cycler (Roche, Basel, Switzerland). Briefly, total-RNA was extracted from each cell line with the Isogen reagent (Nippon Gene, Tokyo, Japan) and 0.1 *μ*g of total-RNA was then reverse transcribed to single-strand cDNA using the RverTra Ace^®^ qPCR RT Kit (Toyobo, Osaka, Japan). Aliquots of the cDNA preparations were subjected to qPCR analysis using SYBR^®^ Premix Ex Taq™ (Takara Bio, Shiga, Japan) to quantify the expression of each target gene and of the internal standard β*-actin* (GenBank Accession no. NM_001101.3) using Light Cycler. The primer pairs used were from the QuantiTect^®^ Primer Assay (Qiagen, Valencia, CA, USA) or were Takara Perfect Real Time Primers (Takara Bio). The results of all assays were checked with the melting curves to confirm the presence of single PCR products.

### Plasmid constructs and transfection studies

The p21^waf1/cip1^ gene was isolated from parental NALM-6 cells by PCR using KOD plus DNA polymerase (Toyobo) and was cloned into pcDNA 3.1 Directional TOPO^®^ Expression vector (Invitrogen), according to the protocol supplied by the manufacturer. Briefly, the sequences of the primers used were 5′-CACCATGTCAGAACCGGCTGGGGATG-3′ for the upstream primer (sense strand, residues 126–147), and 5′-TTAGGGCTTCCTCTTGGAGAAGATCAGC-3′ for the downstream primer (antisense strand, residues 593–620), both of which were synthesized by Operon (Tokyo, Japan), and which yielded a product of about 0.5 kb. PCR amplification was performed for 1 cycle at 94°C for 2 min, which was immediately followed by 30 cycles of 15 sec at 94°C (dissociation), 30 sec at 61°C (primer annealing), and 2 min at 68°C (extension). The PCR products were resolved by electrophoresis on a 1% agarose gel and the gel was stained with ethidium bromide and imaged. The identity of the PCR product was confirmed by direct sequence analysis. The open reading frame of *p21^waf1/cip1^* that was generated by PCR was then inserted into the pcDNA 3.1 expression vector. The obtained cDNA sequence was analyzed and confirmed to be identical with the reported sequence. Empty vectors were used as controls in the experiments.

NALM-6/SP-B or K562 cells were transfected with the pcDNA 3.1 plasmid DNA alone (Empty) or with pcDNA 3.1 containing *p21^waf1/cip1^* cDNA, using the Neon™ Transfection System (Invitrogen) according to the instructions provided by the manufacturer. Briefly, 70–90% confluent cells were harvested and then washed twice with PBS. Subsequently, 2×10^5^ cells were transfected with 1 *μ*g of plasmid in a volume of 10 *μ*l using a Neon tip and electroporation. The conditions of electroporation were: 1,325 V for K562 cells or 1,410 V for NALM-6/SP-B cells, a pulse length of 10 msec, and a total of 3 pulses. The cells were then cultured for 18 h without antibiotics and used for the cytotoxicity assay.

### Western blotting

Changes in the expression level of the p21^waf1/cip1^ protein were detected using western blotting as described in our previous report (18). All antibodies used were purchased from Cell Signaling Technology Inc. (Beverly, MA, USA).

### Statistical analysis

Statistical analysis of the results was performed using a one-way analysis of variance (ANOVA) followed by the Williams’ type multiple comparison test or a Bonferroni test among multiple groups. A p-value of <0.05 was considered statistically significant.

## Results

### Characteristics of SP-B-resistant NALM-6 cells

**Figure 1:**
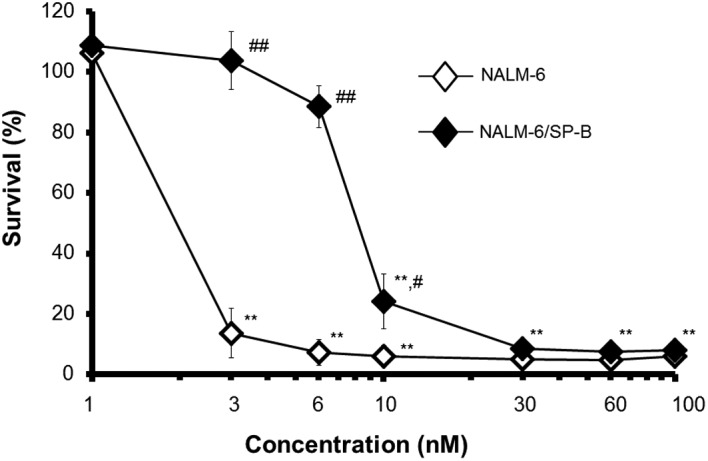
Cytotoxicity of SP-B towards NALM-6 (parent) cells or NALM-6/SP-B (resistant) cells over 72 h. Cells were incubated with the indicated concentrations of SP-B for 72 h, following which cell survival was assayed using the MTT assay. Survival (%) was calculated relative to the control (SP-B vehicle). The results are means ± SEM of three individual studies. ^**^p<0.01 compared with control. ^#^p<0.05, ^##^p<0.01 compared with parental NALM-6 cells.

**Figure 2:**
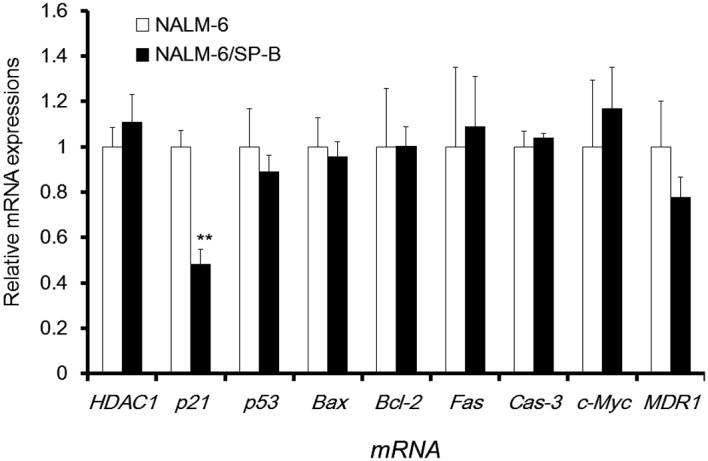
Comparison of the expression of representative mRNA in NALM-6 (parent) and NALM-6/SP-B (resistant) cells. Expression of the indicated mRNA was quantified using qPCR. Changes in mRNA expression are expressed as fold change in mRNA level relative to the mRNA expression of NALM-6 cells. ^**^p<0.01 compared to expression in NALM-6 cells. The results are means ± SEM of three individual studies.

We first established an SP-B-resistant cell line termed NALM-6/SP-B. We had previously calculated that 50% cell growth inhibition (IC_50_) of parental NALM-6 cells by SP-B after 24 h of culture occurred at an SP-B concentration of 5.2 nM (8). We therefore aimed to develop NALM-6 cells that were resistant to SP-B concentrations of up to 6 nM. Whereas SP-B was cytotoxic towards the parental cells at concentrations of SP-B above 1 nM following incubation for 72 h, SP-B did not induce any significant decrease in cell survival of NALM-6/SP-B cells, even at SP-B concentrations of up to 6 nM (Fig. 1). As indicated in Table I, the IC_50_ of SP-B for NALM-6/SP-B cells was 5.9-fold higher than that for parental cells. We additionally compared the effect of other HDIs such as FK228, TSA and NaB, as well as the effect of several typical chemotherapeutic agents, on the growth of NALM-6 and NALM-6/SP-B cells. Although NALM-6/SP-B cells were more resistant to FK228 than the parental cells (4.8-fold higher resistance), the parental and resistant cells showed similar susceptibility to the effects of the other HDIs and of paclitaxel (Taxol; TAX). NALM-6/SP-B cells were slightly more resistant to the P-gp substrates doxorubicin (DOX) and vincristine (VCR) than the parental cells, but this increased resistance was not statistically significant. To further ascertain the characteristics of NALM-6/SP-B cells, we compared the mRNA expression of 9 genes related to cellular growth or apoptosis, including *HDAC1*, *p21^waf1/cip1^*, *p53*, *Bax*, *Bcl-2*, *Fas*, *Cas-3*, *c-Myc*, and *MDR1* in NALM-6/SP-B and control NALM-6 cells using qPCR (Fig. 2). The mRNA expression of *p21^waf1/cip1^* was significantly decreased in NALM-6/SP-B cells to less than half the level of parental cells, but the mRNA expression level of the other genes showed little difference between the two cell types. Thus, downregulation of *p21^waf1/cip1^* appeared to be a characteristic of NALM-6/SP-B cells that is important for their resistance to SP-B.

### The introduction of exogenous p21^waf1/cip1^ leads to the reversal of SP-B resistance

**Figure 3:**
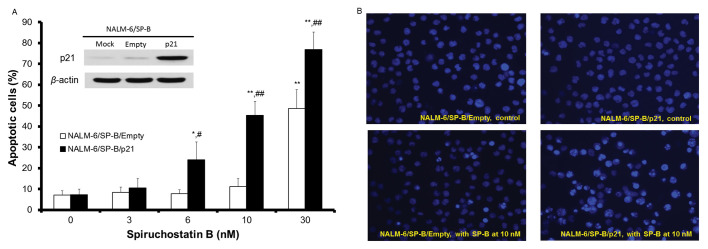
Effect of overexpressed p21^waf1/cip1^ on apoptosis of NALM-6/SP-B cells. NALM-6/SP-B cells were transfected with empty vector (Empty or NALM-6/SP-B/Empty), with a p21^waf1/cip1^ expression vector (p21 or NALM-6/SP-B/p21) or without vector (Mock). (A, inset) p21^waf1/cip1^ protein expression was analyzed by western blotting using β-actin as a loading control. A representative blot of three independent experiments is shown. (A, bar graph) The percentage of apoptotic cells within each group was calculated following staining of cell nuclei with H33342. Apoptotic nuclei were defined by chromatin condensation and partition into multiple bodies. White bars, NALM-6/SP-B/Empty; black bars, NALM-6/SP-B/p21. The results are means ± SEM of three individual studies. ^*^p<0.05, ^**^p<0.01 compared with the respective control. ^#^p<0.05, ^##^p<0.01 compared with NALM-6/SP-B/Empty. (B) Representative photographs of control and SP-B-treated cells whose nuclei were stained with H33342 (magnification, ×400).

Fig. 2 shows that, of the mRNAs expression tested, only *p21^waf1/cip1^* mRNA expression was decreased in NALM-6/SP-B cells. We therefore next constructed a p21^waf1/cip1^ expression vector, and attempted to reverse the SP-B resistance of NALM-6/SP-B cells by transfection of this vector. As shown in the inset to Fig. 3A, the p21^waf1/cip1^ protein was strongly expressed in NALM-6/SP-B cells 24 h after transfection with the p21^waf1/cip1^ expression vector. Overexpression of p21^waf1/ cip1^ was maintained following incubation for 48 h, there was little change of in cell viability, cell cycle arrest or induction of apoptosis of p21^waf1/cip1^ overexpressing NALM-6/SP-B (NALM-6/SP-B/p21) cells compared to NALM-6/SP-B cells (data not shown). These results suggest that introduction of exogenous p21^waf1/cip1^ alone is not sufficient to critically affect cell growth or the induction of apoptosis in NALM-6/SP-B cells. Although apoptosis of NALM-6/SP-B/Empty (transfected with empty control vector) cells was not induced by SP-B at SP-B concentrations of 10 nM or less over a 24-h incubation, apoptosis of NALM-6/SP-B/p21 cells was induced by SP-B at concentrations starting from 6 nM and there was a concentration-dependent increase in NALM-6/SP-B/p21 cell apoptosis between 6 and 30 nM of SP-B. Incubation with 10 nM SP-B for 24 h induced morphological changes typical of apoptosis such as chromatin condensation and apoptotic bodies in NALM-6/SP-B/p21 cells, but induced little change in the morphology of NALM-6/SP-B/Empty cells (Fig. 3B). The percentage cell survival of NALM-6/SP-B/Empty and NALM-6/SP-B/p21 cells following incubation for 48 h with SP-B at concentrations of 3, 6, 10 and 30 nM was 99.8, 91.1, 88.4 and 10.2%, and 73.3, 62.7, 47.5 and 3.4%, respectively. These data indicate that induction of exogenous p21^waf1/cip1^ expression reversed the SP-B resistance of the NALM-6/SP-B cells.

### The introduction of exogenous p21^waf1/cip1^ enhances the susceptibility of another leukemia cell line to SP-B-induced apoptosis

**Figure 4:**
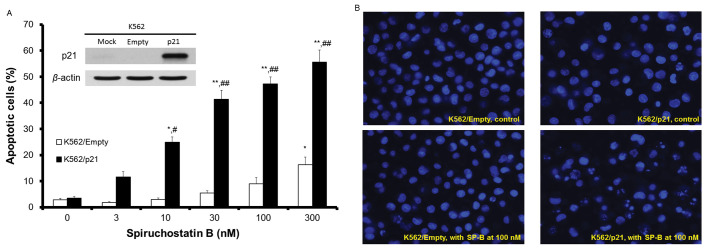
Effect of overexpressed p21^waf1/cip1^ on apoptosis of K562 cells. K562 cells were transfected with empty vector (Empty or K562/Empty), with a p21^waf1/cip1^ expression vector (p21 or K562/p21) or without vector (Mock). (A, inset) p21^waf1/cip1^ protein expression was analyzed by western blotting using β-actin as a loading control. A representative blot of three independent experiments is shown. (A, graph) The percentage of apoptotic cells within each group was calculated following staining of cell nuclei with H33342. Apoptotic nuclei were defined by chromatin condensation and partition into multiple bodies. White bars, K562/Empty; black bars, K562/p21. The results are means ± SEM of three individual studies. ^*^p<0.05, ^**^p<0.01 compared with the respective control. ^#^p<0.05, ^##^p<0.01 compared with K562/Empty. (B) Representative photographs of control and SP-B-treated cells whose nuclei were stained with H33342 (magnification, ×400).

Our previous results indicated that the human erythroleukemia cell line K562 was considerably less susceptible to SP-B than typical leukemia cell lines (8). Thus, the IC_50_ of SP-B for K562 cells was more than 100-fold higher than that for the parental NALM-6 cells. To determine whether introduction of exogenous p21^waf1/cip1^ expression improves the susceptibility of leukemia cells to SP-B, we transfected K562 cells with p21^waf1/cip1^ and analyzed its effect on the susceptibility of these cells to apoptosis induced by SP-B (Fig. 4A and B). As shown in the inset to Fig. 4A, the p21^waf1/cip1^ protein was expressed in K562 cells 24 h after p21^waf1/cip1^ transfection (K562/p21). Following SP-B treatment of K562 control cells (transfected with empty vector, K562/Empty), there was no significant induction of apoptosis by SP-B compared to non-treated cells at SP-B concentrations of up to 100 nM, although apoptosis of these cells was increased at an SP-B concentration of 300 nM. Apoptosis was induced by SP-B in K562/p21 cells in an SP-B concentration-dependent manner and apoptosis of these cells was significantly increased compared to K562/Empty cells at all SP-B concentrations tested between 10 and 300 nM (Fig. 4A). The percentage cell survival of K562/Empty or K562/p21 cells following incubation for 48 h with SP-B at concentrations of 3, 10, 30, 100 and 300 nM was 102.8, 98.4, 87.4, 72.8 and 28.4%, and 85.2, 79.9, 38.2, 19.1 and 13.8%, respectively. Thus, expression of exogenous p21^waf1/cip1^ enhanced the susceptibility of K562 cells to SP-B.

## Discussion

Our previous report showed that SP-B is the most potent HDI for a typical human leukemia cell line and further demonstrated that the expression of p21^waf1/cip1^ plays a pivotal role in SP-B induction of apoptosis and in the resulting anti-tumor activity of SP-B (8). In the present study, we developed and characterized SP-B-resistant NALM-6 cells, and, by comparing SP-B-resistant and -susceptible leukemia cells, showed that the introduction of exogenous p21^waf1/cip1^ into cells reversed their SP-B resistance and enhanced their susceptibility to SP-B.

The SP-B-resistant NALM-6 cells we established were also resistant to FK228 (Table I). All previously established FK228-resistant cells are highly resistant to FK228 compared with parental cells and this resistance depends on increased expression of the P-gp protein that is encoded by the *MDR1* gene (10–12). These previously established FK228-resistant cells acquired drug resistance within a short period (continuous culture for 1 to 3 months) compared with the SP-B-resistant cells that we established (continuous culture for almost 6 months). The SP-B concentration and the culture period necessary for acquisition of resistance to SP-B in this study suggest that it was more difficult to achieve SP-B resistance than FK228 resistance. Furthermore, SP-B resistance had little effect on *MDR1* mRNA expression in NALM-6 cells. However, despite the fact that *MDR1* mRNA expression of NALM-6/SP-B was nearly the same as that of parental cells (Fig. 2), the NALM-6/SP-B cells displayed lower susceptibility to DOX and VCR, which are better substrates for P-gp, than the parental cells (Table I). A previous DNA microarray analysis study indicated that the level of *p21^waf1/cip1^* is significantly decreased in DOX-resistant acute myelocytic leukemia cells (19). This result suggests that a decline in *p21^waf1/ cip1^* contributes to lower sensitivity to DOX. Our data further showed that NALM-6/SP-B cells are slightly more resistant to VCR than parental cells, but show similar resistance to TAX. Both VCR and TAX are M-phase-specific drugs that stabilize microtubules. A previous study indicated that cytotoxicity and apoptosis induced by these agents were potentiated by overexpression of p21^waf1/cip1^ in sarcoma cells (20). In contrast, enforced expression of p21^waf1/cip1^ in leukemia cells attenuated TAX-mediated apoptosis (21). Thus, the involvement of p21^waf1/cip1^ in the action of M-phase-specific drugs may be cell specific. Yamada *et al*(11), using cDNA microarray analysis, showed that gene expression of *p21^waf1/cip1^* increased in FK228-resistant KU812 cells established from a patient with myeloid leukemia in blastic crisis. Unexpectedly, in our study, NALM-6/SP-B showed decreased *p21^waf1/cip1^* mRNA expression compared with parental cells (Fig. 2). We propose that the underlying mechanism is as follows: SP-B has a stronger effect on p21^waf1/cip1^ expression than other HDIs (8); therefore, continuous exposure of NALM-6 parental cells to SP-B might lead to counteraction of HDI effects on p21^waf1/cip1^ expression, resulting in downregulation of p21^waf1/cip1^ mRNA expression. Such a decrease may not occur following continuous exposure of cells to other HDIs, which have a lower effect on histone deacetylases, and therefore on p21^waf1/cip1^ expression.

As shown in Fig. 2, although endogenous *p21^waf1/cip1^* mRNA expression was decreased in NALM-6 cells that had acquired SP-B resistance, neither the mRNA expression of *HDAC1* nor that of other transcription factors such as *p53* or *c-Myc*, or of apoptosis-related genes showed any change. This *HDAC1* result is in contrast to HL-60 human promyelocytic leukemia cells that are resistant to HDIs, which have been shown to possess significantly higher levels of HDAC1 than parental cells (22). Several other studies in which p53 was assayed as a representative apoptosis-related transcription factor indicated that HDI-induced apoptosis involves the p53 pathway (23,24). In contrast, it has also been shown that HDI induces apoptosis through a p53-independent pathway in leukemia cells (25,26). Our result suggests that the SP-B resistance of NALM-6 cells does not involve p53 expression. Indeed, SP-B did not change p53 expression and pifithrin-α, an inhibitor of p53, and did not affect SP-B-induced apoptosis in either parental NALM-6 or NALM-6/SP-B cells (data not shown). The mRNA expression of the proto-oncogene *c-Myc*, in addition to that of *p21^waf1/cip1^*, has been shown to be a useful indicator of the anti-tumor activity of FK228 (27). Moreover, cDNA microarray analysis of a human acute T cell leukemia cell line (CEM) has shown that HDIs regulate not only *c-Myc*, but also other genes such as *Bax*, *Bcl-2* and *Cas-3* that are involved in the intrinsic apoptotic pathway (28). Another study has proposed that FR901228 (also called FK228) can upregulate the Fas system in osteosarcoma cells, resulting in caspase activation (29). However, as mentioned above, no significant change in the mRNA levels of these previously reported signals was observed in the SP-B-resistant NALM-6 cells. Here, we demonstrated that the decrease in *p21^waf1/cip1^* mRNA expression was a unique characteristic of the SP-B-resistant NALM-6 cells. This is how induction of apoptosis by SP-B in NALM-6/SP-B was reversed, by the introduction of exogenous p21^waf1/cip1^ expression, which compensated for the decrease in endogenous p21^waf1/cip1^ in the resistant cells (Fig. 3).

Recent studies have shown that p21^waf1/cip1^ can mediate both pro- and anti-apoptotic functions in response to anti-tumor agents depending on the cell type and the cellular context (30). As shown in Fig. 4A and B, overexpressed p21^waf1/cip1^ enhanced SP-B-induced apoptosis in K562 cells. This result suggests that p21^waf1/cip1^ has pro-apoptotic functions in K562 cells. Furthermore, the enhancing effect of exogenous p21^waf1/cip1^ on the susceptibility of K562 cells to SP-B-induced apoptosis was stronger than its effect on the reversal of the resistance of NALM-6/SP-B cells to SP-B (Fig. 3). In other words, overexpressed p21^waf1/cip1^ could not completely reverse the resistance of NALM-6/SP-B so that it had the same level of SP-B susceptibility as the parental cells. Most studies on induction of apoptosis by p21^waf1/cip1^ were reported using cells that expressed a non-functional p53 mutant (30). It has already been reported that K562 cells harbor mutated p53 (31,32), and that NALM-6 cells express wild-type p53 (18,32). The ability of overexpressed p21^waf1/cip1^ to enhance SP-B-induced apoptosis is assumed to depend on p53 status. Elucidation of the relationship between p53 status and p21^waf1/cip1^ expression may lead to the establishment of effective SP-B chemotherapy for leukemia patients.

In conclusion, NALM-6 cells that were resistant to SP-B showed a unique decrease in the mRNA expression of *p21^waf1/cip1^*. The introduction of exogenous p21^waf1/cip1^ not only reversed the SP-B resistance of these NALM-6 cells, but also enhanced the susceptibility of K562 cells to apoptosis induced by SP-B. Based on these results, introduction of exogenous p21^waf1/cip1^ into tumor cells in a clinical setting should improve tumor resistance to SP-B or may lead to successful chemotherapy using SP-B. Our findings may be useful when establishing a therapeutic strategy using SP-B.

## Figures and Tables

**Table I:** The cytotoxicity of anti-cancer drugs towards NALM-6 and NALM-6/SP-B.

	IC_50_		
Drugs (concentration)	NALM-6	NALM-6/SP-B	Index
SP-B (nM)	1.6 (1.03–2.73)	9.9 (5.98–16.55)	×5.9
FK228 (nM)	2.1 (1.31–3.54)	10.3 (6.48–16.36)	×4.8
TSA (nM)	33.3 (23.13–48.08)	36.5 (24.08–55.45)	×1.1
NaB (mM)	0.86 (0.668–1.120)	1.3 (0.88–2.10)	×1.6
DOX (nM)	15.8 (9.99–25.23)	42.6 (24.84–73.06)	×2.7
VCR (nM)	3.4 (2.10–5.53)	8.9 (5.53–14.43)	×2.6
TAX (nM)	4.5 (2.26–9.13)	5.4 (2.85–10.45)	×1.2

The cells were treated with the anti-cancer drugs for 72 h and subsequently cytotoxicity was assessed using the MTT assay. Data were calculated relative to each control group and are expressed as the concentration which results in 50% cell growth inhibition (IC_50_). The range of values obtained is shown in brackets under each result.

## References

[1] Narita K, Kikuchi T, Watanabe K (2009). Total synthesis of the bicyclic depsipeptide HDAC inhibitors spiruchostatins A and B, 5″-epi-spiruchostatin B, FK228 (FR901228) and preliminary evaluation of their biological activity. Chemistry.

[2] Ocker M, Schneider-Stock R (2007). Histone deacetylase inhibitors: signalling towards p21cip1/waf1. Int J Biochem Cell Biol.

[3] Xiong Y, Zhang H, Beach D (1992). D type cyclins associate with multiple protein kinases and the DNA replication and repair factor PCNA. Cell.

[4] Han JW, Ahn SH, Kim YK (2001). Activation of p21(WAF1/Cip1) transcription through Sp1 sites by histone deacetylase inhibitor apicidin: involvement of protein kinase C. J Biol Chem.

[5] Fournel M, Trachy-Bourget MC, Yan PT (2002). Sulfonamide anilides, a novel class of histone deacetylase inhibitors, are anti-proliferative against human tumors. Cancer Res.

[6] Archer SY, Meng S, Shei A, Hodin RA (1998). p21(WAF1) is required for butyrate-mediated growth inhibition of human colon cancer cells. Proc Natl Acad Sci USA.

[7] Sambucetti LC, Fischer DD, Zabludoff S (1999). Histone deacetylase inhibition selectively alters the activity and expression of cell cycle proteins leading to specific chromatin acetylation and antiproliferative effects. J Biol Chem.

[8] Kanno SI, Maeda N, Tomizawa A, Yomogida S, Katoh T, Ishikawa M (2012). Involvement of p21^waf1/cip1^ expression in the cytotoxicity of the potent histone deacetylase inhibitor spiruchostatin B towards susceptible NALM-6 human B cell leukemia cells. Int J Oncol.

[9] Szakacs G, Annereau JP, Lababidi S (2004). Predicting drug sensitivity and resistance: profiling ABC transporter genes in cancer cells. Cancer Cell.

[10] Xiao JJ, Huang Y, Dai Z (2005). Chemoresistance to depsipeptide FK228 [(E)-(1S,4S,10S,21R)-7-[(Z)-ethylidene]-4,21-diisopropyl-2-oxa-12,13-dithia-5,8,20,23-tetraazabicyclo[8,7,6]-tricos-16-ene-3,6,9,22-pentanone] is mediated by reversible MDR1 induction in human cancer cell lines. J Pharmacol Exp Ther.

[11] Yamada H, Arakawa Y, Saito S, Agawa M, Kano Y, Horiguchi-Yamada J (2006). Depsipeptide-resistant KU812 cells show reversible P-glycoprotein expression, hyper-acetylated histones, and modulated gene expression profile. Leuk Res.

[12] Matsubara H, Watanabe M, Imai T (2009). Involvement of extracellular signal-regulated kinase activation in human osteosarcoma cell resistance to the histone deacetylase inhibitor FK228 [(1S,4S,7Z,10S,16E,21R)-7-ethylidene-4,21-bis(propan-2-yl)-2-oxa-12,13-dit hia-5,8,20,23-tetraazabicyclo[8.7.6] tricos-16-ene-3,6,9,19,22-pentone]. J Pharmacol Exp Ther.

[13] Imesch P, Dedes KJ, Furlato M, Fink D, Fedier A (2009). MLH1 protects from resistance acquisition by the histone deacetylase inhibitor trichostatin A in colon tumor cells. Int J Oncol.

[14] Dedes KJ, Dedes I, Imesch P, von Bueren AO, Fink D, Fedier A (2009). Acquired vorinostat resistance shows partial cross-resistance to ‘second-generation’ HDAC inhibitors and correlates with loss of histone acetylation and apoptosis but not with altered HDAC and HAT activities. Anticancer Drugs.

[15] Takizawa T, Watanabe K, Narita K, Oguchi T, Abe H, Katoh T (2008). Total synthesis of spiruchostatin B, a potent histone deacetylase inhibitor, from a microorganism. Chem Commun (Camb).

[16] Kanno S, Kakuta M, Kitajima Y (2007). Preventive effect of trimidox on oxidative stress in U937 cell line. Biol Pharm Bull.

[17] Kanno S, Hiura T, Ohtake T (2007). Characterization of resistance to cytosine arabinoside (Ara-C) in NALM-6 human B leukemia cells. Clin Chim Acta.

[18] Kanno S, Higurashi A, Watanabe Y, Shouji A, Asou K, Ishikawa M (2004). Susceptibility to cytosine arabinoside (Ara-C)-induced cytotoxicity in human leukemia cell lines. Toxicol Lett.

[19] Song JH, Choi CH, Yeom HJ, Hwang SY, Kim TS (2006). Monitoring the gene expression profiles of doxorubicin-resistant acute myelocytic leukemia cells by DNA microarray analysis. Life Sci.

[20] Li W, Fan J, Banerjee D, Bertino JR (1999). Overexpression of p21(waf1) decreases G2-M arrest and apoptosis induced by paclitaxel in human sarcoma cells lacking both p53 and functional Rb protein. Mol Pharmacol.

[21] Ahmed W, Rahmani M, Dent P, Grant S (2004). The cyclin-dependent kinase inhibitor p21(CIP1/WAF1) blocks paclitaxel-induced G2M arrest and attenuates mitochondrial injury and apoptosis in p53-null human leukemia cells. Cell Cycle.

[22] Fiskus W, Rao R, Fernandez P (2008). Molecular and biologic characterization and drug sensitivity of pan-histone deacetylase inhibitor-resistant acute myeloid leukemia cells. Blood.

[23] Bandyopadhyay D, Mishra A, Medrano EE (2004). Overexpression of histone deacetylase 1 confers resistance to sodium butyrate-mediated apoptosis in melanoma cells through a p53-mediated pathway. Cancer Res.

[24] Condorelli F, Gnemmi I, Vallario A, Genazzani AA, Canonico PL (2008). Inhibitors of histone deacetylase (HDAC) restore the p53 pathway in neuroblastoma cells. Br J Pharmacol.

[25] Vrana JA, Decker RH, Johnson CR (1999). Induction of apoptosis in U937 human leukemia cells by suberoylanilide hydroxamic acid (SAHA) proceeds through pathways that are regulated by Bcl-2/Bcl-XL, c-Jun, and p21CIP1, but independent of p53. Oncogene.

[26] Insinga A, Monestiroli S, Ronzoni S (2005). Inhibitors of histone deacetylases induce tumor-selective apoptosis through activation of the death receptor pathway. Nat Med.

[27] Sasakawa Y, Naoe Y, Inoue T (2003). Effects of FK228, a novel histone deacetylase inhibitor, on tumor growth and expression of p21 and c-myc genes in vivo. Cancer Lett.

[28] Peart MJ, Smyth GK, van Laar RK (2005). Identification and functional significance of genes regulated by structurally different histone deacetylase inhibitors. Proc Natl Acad Sci USA.

[29] Imai T, Adachi S, Nishijo K (2003). FR901228 induces tumor regression associated with induction of Fas ligand and activation of Fas signaling in human osteosarcoma cells. Oncogene.

[30] Liu S, Bishop WR, Liu M (2003). Differential effects of cell cycle regulatory protein p21(WAF1/Cip1) on apoptosis and sensitivity to cancer chemotherapy. Drug Resist Updat.

[31] Law JC, Ritke MK, Yalowich JC, Leder GH, Ferrell RE (1993). Mutational inactivation of the p53 gene in the human erythroid leukemic K562 cell line. Leuk Res.

[32] Filippini G, Griffin S, Uhr M (1998). A novel flow cytometric method for the quantification of p53 gene expression. Cytometry.

